# Minimal Important Difference (MID) of two commonly used outcome measures for foot problems

**DOI:** 10.1186/1757-1146-3-7

**Published:** 2010-05-14

**Authors:** Karl B Landorf, Joel A Radford, Susie Hudson

**Affiliations:** 1 Department of Podiatry, Faculty of Health Sciences, La Trobe University, Bundoora, Victoria, 3086, Australia; 2 Musculoskeletal Research Centre, Faculty of Health Sciences, La Trobe University, Bundoora, Victoria, 3086, Australia; 3 Department of Podiatry, University of Western Sydney, Penrith South, New South Wales, 1001, Australia

## Abstract

**Background:**

The Visual Analogue Scale (VAS) and the Foot Health Status Questionnaire (FHSQ) are two commonly used outcome measures for evaluating foot health. This study aimed to calculate the *Minimal Important Difference *(MID) of the VAS and the FHSQ.

**Methods:**

184 participants with plantar heel pain were recruited from the general public to take part in two randomised trials (92 participants in each trial) that studied the effectiveness of two conservative interventions for plantar heel pain. Data from these participants were used to calculate the MIDs of the VAS and the FHSQ. An anchor-based method was used to calculate the MIDs. Two distinct types of pain were investigated for the VAS: average pain and first-step pain. All four domains of the FHSQ were investigated: foot pain, foot function, footwear and general foot health.

**Results:**

The MID for the VAS using the anchor-based approach was -8 mm (95% CI: -12 to -4) for average pain and -19 mm (95% CI: -25 to -13) for first-step pain on the 100 mm VAS. The MID for the FHSQ was 13 points (95% CI: 6 to 19) for pain and 7 points (95% CI: 1 to 13) for function. The MID for the footwear domain of the FHSQ was -2 points (95% CI: -8 to 4) and 0 points (95% CI: -7 to 6) for the general foot health domain of the FHSQ.

**Conclusion:**

The results of this study provide additional evidence for MID values of the VAS and the FHSQ for plantar heel pain. This is important for clinicians and researchers as it provides a greater understanding of how much improvement is required by a patient before a minimal, worthwhile change is experienced. The calculated MIDs will also assist researchers with prospective sample size calculations.

## Background

Health outcome assessment is an important component of health care. Outcome measures are primarily used to objectively detect change in a patient's health status in response to an intervention [1]. They may also be used to measure a patient's health status at a specific point in time [2].

As outcome measurement has developed, there has been a growing appreciation of the patient's perspective of their disease and their preferences for treatment [3]. Outcome measures completed by the patient (i.e. self-reported) are now commonly referred to as patient-reported outcomes [4]. Patient-reported outcome measures assist clinicians' understanding of the effects of the disease on a patient's capabilities, functioning and symptoms [5]; that is the effect on their health status (or health-related quality of life).

If a significant change in health status occurs after an intervention has been implemented a patient should be able to perceive this change and regard it as important [6]. Schunemann and Guyatt [7] have suggested the term *minimal important difference *(MID) be used to represent this change. The MID has been defined as "the smallest difference in score in the domain of interest which patients perceive as beneficial and which would mandate, in the absence of troublesome side effects and excessive cost, a change in the patient's management" (p. 408) [6]. The MID denotes the smallest change in health status that is regarded as important by the patient. Accordingly, it can be used to determine if a patient has experienced an important or worthwhile change in their health status as a result of an intervention. From a research perspective, the MID is fundamental for calculating sample size for trials in the future and can provide meaning to results of clinical trials that have already been conducted [8].

There are many patient-reported outcome measures that are used for conditions that affect the foot or ankle. The Visual Analogue Scale (VAS) and the Foot Health Status Questionnaire (FHSQ) are two such outcome measures [9,10]. Clinicians and researchers use the VAS to measure pain severity and pain relief when an intervention is implemented. The FHSQ - a more complex health status measure - was developed to measure health-related quality of life with respect to foot health [11]. Currently, there has only been one study that has investigated the MID values for the VAS and the FHSQ in research relating to foot health [12]. Although this study used an appropriate technique (an anchor-based method), it was not originally designed to calculate MIDs. In addition, the study used a relatively imprecise 4-point Likert Scale to determine which participants experience a minimally important change. To provide greater precision when estimating the MID, it has been suggested that a Likert scale with up to 15-points be used [6].

Therefore, this study aimed to calculate with greater precision the MIDs of two commonly used outcome measures for foot-related research: the Visual Analogue Scale and the Foot Health Status Questionnaire.

## Methods

Data for this study were taken from two randomised trials evaluating conservative treatments for plantar heel pain. Both of these trials used similar methodologies - the same general protocol and outcome measures - which have been reported in detail (including participant characteristics) elsewhere [13,14]. Relevant data from these trials were extracted for this study to determine the MIDs for the VAS and the FHSQ.

### Participants

Between February and June 2005, 184 participants with plantar heel pain were recruited from the general public to take part in two randomised trials (92 participants in each trial). These research projects studied the effectiveness of two conservative interventions (taping and stretching) for plantar heel pain. Patients who had a clinical diagnosis of plantar heel pain and had suffered symptoms for at least four weeks were invited to participate. People were excluded if they had inflammatory, osseous, metabolic or neurological disorders. They were also excluded if they had received a corticosteroid injection within the past three months, or had a known allergy to adhesive tape if being recruited into the taping trial. Ethical approval was obtained from the relevant institutional ethics committees and all participants gave written informed consent to participate in the original trials.

### Outcome Measures

The outcome measures were assessed after one week in the taping trial and after two weeks in the stretching trial. The VAS was used to measure pain levels [15,16] and the FHSQ to measure foot health status. With respect to the VAS, there were two different types of pain that were being investigated: 'average pain' and 'first-step pain'. With respect to the FHSQ, health status was measured for all four domains: 'pain', 'function', 'footwear' and 'general foot health' [11,17]. Both outcome measures have undergone appropriate validation [17,18].

All the outcomes provided continuous data for each domain on a scale of 0-100. For the VAS, the lower the score the better (i.e. 0 = no pain and 100 = worst pain). In contrast, for the FHSQ the higher the score the better (i.e. 100 = best foot health and 0 = worst foot health).

### Calculation of the MIDs

MIDs were calculated for the VAS average pain and first-step pain, and for the four domains of the FHSQ (pain, function, footwear and general foot health). An anchor-based approach was used to calculate the MIDs. The anchor-based method has been suggested to be the most appropriate method to determine the MID [8].

**Figure 1:**
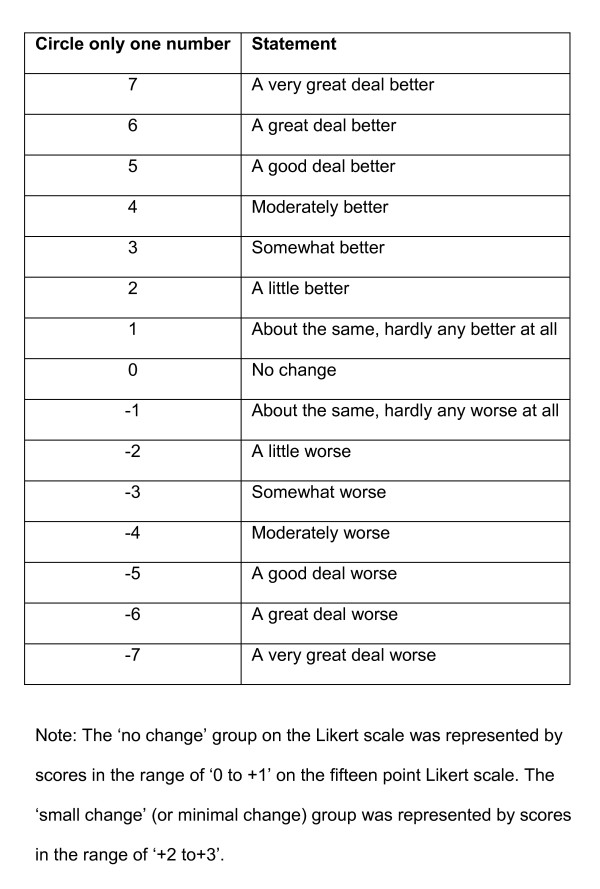
**The 15-point Likert scale used in the study**.

To calculate the MID using an anchor-based approach, a 15-point Likert scale (Figure 1) was used. On the 15-point Likert scale each number represents a different change in health status. The numbers range from minus seven to plus seven. Minus seven represents a change in health status that is 'a very great deal worse', while plus seven indicates a change that is 'a very great deal better'. The value of zero represents 'no change' in health status. For this project participants who answered '+2 and +3' represented a 'small change' and participants who answered '0 and +1' represented 'no change' in health status. The 'no change' group on the Likert scale was represented by scores in the range of '0 to +1' on the fifteen point Likert scale. The 'small change' (or minimal change) group was represented by scores in the range of '+2 to+3'.

**Figure 2:**
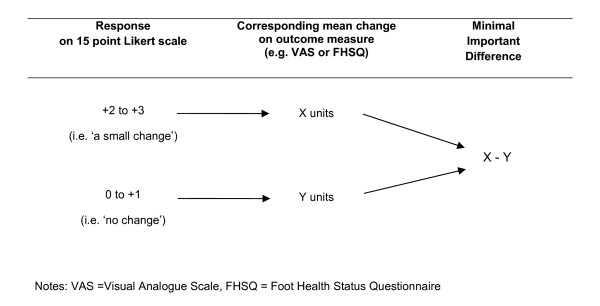
**Method used to calculate the MID using the anchor-based approach**.

To begin the MID calculation, the mean change in both outcome measures (VAS and FHSQ) from baseline for all participants who answered 'no change' and 'a small change' on the 15-point Likert scale were calculated. The mean change on the outcome measures for the participants who answered 'no change' was then subtracted from the mean change in outcome measure for the participants who had experienced 'a small change' to form the MID value (illustrated in Figure 2). This methodology was similar to that used by Landorf and Radford [12]; however they only used a 4-point Likert scale.

Mean change for the outcome measures were calculated using Statistical Program for the Social Sciences (SPSS) version 14.0. To assess precision of the MID estimates, 95% confidence intervals were also calculated using the statistical program Confidence Interval Analysis (Version 2.0.0). As parametric statistics were reported (e.g. mean) all outcome data were checked for normalcy to satisfy the necessary assumptions. Variables demonstrating a distribution that was not normal were then assessed for outliers (i.e. participants that had scores 3 standard deviations from the mean) and those participants were removed from the analysis of that variable to ensure a normal distribution. Three participants in the 'VAS average pain' variable and one participant in the 'FHSQ footwear' variable were removed for this reason.

## Results

The MID values of the anchor-based approach for the VAS and the FHSQ are presented in Table 1. The MID for the VAS average pain was approximately -8 mm (95% CI: -12 to -4) and -19 mm (95% CI: -25 to -13) for first-step pain. The MIDs for the four domains of the FHSQ were approximately 13 points (95% CI: 6 to 19) for pain, 7 points (95% CI: 1 to 14) for function, -2 points (95% CI: -8 to 4) for footwear and 0 points (95% CI: -7 to 6) for general foot health.

**Table 1:** Anchor-based calculations of MID for the VAS and the FHSQ

Outcome	Domain	0 to +1	+2 to +3	MID values	95% CI
Measure		n = 57	n = 40		
VAS	Average	-1.1^§^	-8.9^†^	-7.8	-11.7 to -3.9
	1st Step	-1.9	-20.5	-18.6	-24.6 to -12.6
					
FHSQ	Pain	8.7	21.2	12.5	5.8 to 19.2
	Function	4.8	11.9	7.1	0.7 to 13.4
	Footwear	1.9^‡^	-0.2	-2.1	-8.0 to 3.8
	GFH	8.3	7.9	-0.4	-7.1 to 6.4

Abbreviations: VAS = Visual Analogue Scale, FHSQ = Foot Health Status Questionnaire, MID = Minimal Important Difference, GFH = General Foot Health, CI = Confidence Interval.

Note: Three variables were initially not normally distributed, so outliers that were 3SDs from the mean were removed to ensure normalcy. Accordingly, some variables had a lower sample size due to this, which the following symbols represent: ^§^n = 55, ^‡^n = 56, ^†^n = 39.

## Discussion

The VAS and the FHSQ are two outcome measures that are commonly used in foot-related research. With respect to plantar heel pain, the MIDs for the VAS and the FHSQ have only been estimated once previously [12]. This previous study calculated the MID of the VAS when pain was 'at its worst' and only calculated MIDs for three domains of the FHSQ (pain, function and general foot health). A limitation to this study was that a relatively blunt 4-point Likert scale method was used instead of a more precise method (i.e. a 15-point Likert scale) to calculate the MIDs for the VAS and the FHSQ. The present study aimed to improve on this methodology thereby attempting to provide more precise estimates of the MIDs.

For the VAS, the MID was calculated for both average pain and for first-step pain. Using the anchor-based approach the MID for the VAS average pain was found to be -8 mm (i.e. an improvement of 8 mm on the 100 mm VAS) and -19 mm (i.e. an improvement of 19 mm on the 100 mm VAS) for first-step pain. First-step pain is the pain experienced after a long period of non-weightbearing (e.g. upon first stepping out of bed in the morning) and is a common complaint of patients with plantar heel pain. This indicates that on a 100 mm VAS, a reduction of 8 mm for average pain and 19 mm for first-step pain was needed for a patient to recognise a worthwhile minimal change in their pain level. The MID value for average pain is comparable to our previous research that suggested a MID value for the VAS was 9 mm for people with plantar heel pain [12]. Interestingly, research in the emergency medicine literature indicates that the MID for the VAS when used in an emergency department ranges between 9 and 13 mm [19-21].

Compared to average pain, first-step pain appears to require a greater improvement before a minimally important change is detected by patients with plantar heel pain (19 mm for first-step pain versus 8 mm for average pain). This may be due to VAS scores for first-step pain being initially much higher compared to average pain, and therefore, a greater amount of pain relief is required to be deemed important or worthwhile. However, such a difference has not been found when comparing severity of pain being experienced in an emergency medicine department [21].

With respect to the FHSQ, we found a MID of 13 points for pain (i.e. an improvement in pain of 13 points) and 7 points for function (i.e. an improvement in function of 7 points). These are the estimated values for a minimally important change that can be detected by patients. These values are similar to our previous study (also determining the MIDs for plantar heel pain) which determined the MID values for the pain and function domains of the FHSQ to be 14 points for pain and 7 points for function [12].

The current study was the first to calculate the MID for the footwear domain of the FHSQ. The very small negative value of -2 points (i.e. a worsening) for footwear may indicate that the four domains of the FHSQ capture different constructs of health status. A similar result was found for the general foot health domain of the FHSQ where a MID of 0 points was estimated. Therefore, this study of participants with plantar heel pain may have simply demonstrated that the domains of footwear and general foot health are independent of both pain and function, something that would appear intuitive and which may further validate the instrument's ability to disaggregate different components that contribute to health status. However, an alternative explanation that cannot be discounted may be that for pathology such as plantar heel pain these sub-scales are not sensitive enough to detect change - this issue has been raised previously for general foot health [22]. Further research is warranted in this area.

The results calculated in this study for the VAS and the FHSQ may need to be used cautiously for other foot pathologies that cause more severe levels of disability and impairment in foot health compared to plantar heel pain. For example, if a patient has severe pain and impairment due to rheumatoid arthritis it may be reasonable to expect that the MIDs for the FHSQ in the pain and function domains would be much larger. That is, higher initial levels of pain and impairment in foot function may require a greater reduction before the patient would consider it to be worthwhile. As such, patients with such pathology may have to experience more improvement than 13 points for pain or 7 points for function on the FHSQ - the MIDs we have calculated for plantar heel pain - before they would consider the change worthwhile.

The MIDs calculated in this study may be used to interpret results of other trials that utilise the VAS and the FHSQ for measuring outcomes in pathology similar to plantar heel pain. For example, if the effect of an intervention is well below 13 points (e.g. a difference of 5 points) in the pain domain of the FHSQ, then the effect of the intervention is most likely too small for patients to detect an important change, regardless of whether the intervention is shown to be statistically significant. This highlights the importance when evaluating an intervention in a clinical trial of considering both clinical significance as well as statistical significance. A statistically significant result may not always indicate that a patient has experienced a clinically worthwhile change.

Findings from this trial may also be used to calculate sample sizes for future trials. Prospective sample size calculations are fundamental to the planning of any clinical trial. By calculating an appropriate sample size, the study will have sufficient statistical power to detect clinically worthwhile changes if they do in fact exist. An underpowered study, may lead to a Type 2 statistical error (i.e. the study concludes that there is no difference between the two groups, when in fact there was a clinically worthwhile difference, but the sample size was insufficient to detect it) [23]. Similar to our previous study [12], and in the absence of evidence to the contrary, we cautiously suggest that our results may be used to calculate appropriate sample sizes for trials investigating interventions for musculoskeletal disorders of the foot that cause a similar level of pain and disability to plantar heel pain.

The results of this study need to be viewed in light of a number of potential limitations. Firstly, for the anchor-based approach the number of participants in the 'no change' group and the 'a little change' group was 57 and 40 respectively. Larger numbers of participants in these groups would lead to greater precision when calculating the MID [8]. The relatively low numbers in these groups was in part due to there being a greater amount of categories available on the 15-point Likert scale (as opposed to the 4-point scale used in our previous study[12]). As a result, the participants were distributed across a greater number of categories; the end result being that the number of participants in each category was relatively less compared to a 4-point Likert scale. Therefore, the width of the 95% confidence intervals calculated for the MID using the 15-point Likert scale were similar to those in the previous study we conducted [12] that only used a 4-point Likert scale. This resulted in no greater precision using the 15-point Likert scale to calculate the MIDs in this study. Accordingly, it would be beneficial in future research to recruit a larger sample size to ensure that there are more participants in the 'small change' and 'no change' categories on the 15-point Likert scale.

Finally, and as discussed previously, the data used from the two trials only assessed conservative treatments for plantar heel pain. Therefore, the MID values may not be able to be generalised to other pathologies and treatments. Ideally, future research will investigate the MIDs for a range of patient-reported outcome measures across a spectrum of pathology.

## Conclusion

This research project calculated the MIDs for the VAS and the FHSQ. The results of this study provide further evidence of the MID values for both outcome measures. The findings will assist in interpreting results from clinical trials that have used the VAS and the FHSQ as outcome measures to evaluate an intervention's effectiveness, particularly for plantar heel pain. Further research would be useful to determine the MIDs for a variety of foot pathologies that cause different levels of pain and disability for patients. With the aforementioned limitations in mind, researchers and clinicians can cautiously use these MID values to assist in determining clinically worthwhile changes in a patient's health status after an intervention has been implemented. Finally, the MID values are fundamental for prospective sample size calculations for clinical trials.

## Competing interests

KBL is Deputy Editor in Chief of the Journal of Foot and Ankle Research. It is journal policy that editors are removed from the peer review and editorial decision making process for the papers that they have co-authored.

## Authors' contributions

KBL and JAR designed the study. JAR was the primary data collector. KBL, SH and JAR and were involved with data analysis and interpretation. All authors read and approved the final version of the manuscript.
